# Computerization of the Work of General Practitioners: Mixed Methods Survey of Final-Year Medical Students in Ireland

**DOI:** 10.2196/42639

**Published:** 2023-03-20

**Authors:** Charlotte Blease, Anna Kharko, Michael Bernstein, Colin Bradley, Muiris Houston, Ian Walsh, Kenneth D Mandl

**Affiliations:** 1 General Medicine and Primary Care Beth Israel Deaconess Medical Center Boston, MA United States; 2 Healthcare Sciences and e-Health Department of Women's and Children's Health Uppsala University Uppsala Sweden; 3 School of Psychology University of Plymouth Plymouth United Kingdom; 4 Department of Behavioral and Social Sciences School of Public Health Brown University Providence, RI United States; 5 Department of Diagnostic Imaging Warren Alpert Medical School Brown University Providence, RI United States; 6 School of Medicine University College Cork Cork Ireland; 7 School of Medicine National University of Ireland Galway Galway Ireland; 8 School of Medicine Trinity College Dublin Dublin Ireland; 9 Dentistry and Biomedical Sciences School of Medicine Queen's University Belfast Ireland; 10 Computational Health Informatics Program Boston Children's Hospital Boston, MA United States

**Keywords:** medical students, medical education, general practitioners, artificial intelligence, machine learning, digital health, technology, tool, medical professional, biomedical, design, survey, COVID-19

## Abstract

**Background:**

The potential for digital health technologies, including machine learning (ML)–enabled tools, to disrupt the medical profession is the subject of ongoing debate within biomedical informatics.

**Objective:**

We aimed to describe the opinions of final-year medical students in Ireland regarding the potential of future technology to replace or work alongside general practitioners (GPs) in performing key tasks.

**Methods:**

Between March 2019 and April 2020, using a convenience sample, we conducted a mixed methods paper-based survey of final-year medical students. The survey was administered at 4 out of 7 medical schools in Ireland across each of the 4 provinces in the country. Quantitative data were analyzed using descriptive statistics and nonparametric tests. We used thematic content analysis to investigate free-text responses.

**Results:**

In total, 43.1% (252/585) of the final-year students at 3 medical schools responded, and data collection at 1 medical school was terminated due to disruptions associated with the COVID-19 pandemic. With regard to forecasting the potential impact of artificial intelligence (AI)/ML on primary care 25 years from now, around half (127/246, 51.6%) of all surveyed students believed the work of GPs will change minimally or not at all. Notably, students who did not intend to enter primary care predicted that AI/ML will have a great impact on the work of GPs.

**Conclusions:**

We caution that without a firm curricular foundation on advances in AI/ML, students may rely on extreme perspectives involving self-preserving optimism biases that demote the impact of advances in technology on primary care on the one hand and technohype on the other. Ultimately, these biases may lead to negative consequences in health care. Improvements in medical education could help prepare tomorrow’s doctors to optimize and lead the ethical and evidence-based implementation of AI/ML-enabled tools in medicine for enhancing the care of tomorrow’s patients.

## Introduction

### Background

According to economists and futurists, traditional health care will become increasingly disintermediated by innovations in digital technology, including advances in artificial intelligence (AI)/machine learning (ML) [1-3]. These views are also held by many AI experts and health care informaticians, many of whom are physicians, who predict that ongoing developments in AI/ML will revolutionize the delivery of health care [4-7]. Moreover, digital innovations and AI/ML-enabled tools already play roles in health care by helping patients to monitor and manage their symptoms, supporting patient triage decisions via chatbots, informing clinical decisions, offering treatment recommendations via clinical decision support tools, and supporting health care resource management [8]. Despite these developments, in surveys, many medical professionals are skeptical about the impact and value of digital and AI/ML tools on their job, with surveyed physicians doubting the scope of technological innovations to replace clinicians in fundamental medical tasks [9-11]. Emerging surveys among students enrolled in a range of health care training programs, including medicine, dentistry, and clinical psychology, also revealed divergent opinions about the impact of AI/ML on their chosen profession, with participants reporting limited formal education on these topics [12-18].

### Objectives

We sought to explore the opinions of final-year medical students in Ireland on the impact of future technology on the job of general practitioners (GPs). We performed a brief scoping review of the literature using the terms “artificial intelligence,” “machine learning,” “education,” and “training” in the search engines of PubMed and Google Scholar, and explored the grey literature. Only a few surveys, which were conducted in Europe, the United States, and South Korea, explored the attitudes of medical or health care students about the encroachment of AI/ML in medicine, and most were single-site studies [12-18]. Our objective was to explore the opinions of final-year medical students across Ireland to obtain a better understanding of their forecasts about the capacity of future technology to fully replace or to partner with physicians in undertaking key components of the work of GPs. In addition, our aim was to explore both students’ longer-term predictions and comparatively shorter-term forecasts (25 years from now) about how technology might impact the work of GPs.

## Methods

### Study Population

Participants in this convenience sample paper-based survey were final-year medical students at 4 of Ireland’s 7 medical schools (after survey administration, in August 2021, a new 8th medical school at the University of Ulster began enrolling students). Using the study team’s contacts, we sought to administer the survey in the country’s 4 geographical provinces. Between April 2019 and March 2020, the anonymous survey was distributed by lecturers after compulsory final-year classes at each institution to increase responses.

### Ethics Approval

Institutional review boards at University College Cork (protocol #2018-188), National University of Ireland Galway (protocol #19-Dec-15), Queen’s University Belfast (protocol #19.28), and University College Dublin (protocol #LS-19-89) approved the study at their respective sites. Participation was voluntary, and all students who decided to participate provided written consent.

### Survey Instrument

The survey (Multimedia Appendix 1) was divided into 5 parts (Sections A to E). Section A requested demographic information. In Section B, the study team replicated and also extended components of a survey instrument originally devised to investigate the views of UK GPs about the potential impact of technology on the primary care profession [9]. The survey by Blease et al [9] formulated a generic list of tasks common to primary care, including “analyze patient information to reach diagnoses,” “analyze patient information to predict the likely course of the patient’s illness,” “evaluate when to refer patients to other health professionals,” “formulate personalized treatment plans,” “provide empathic care to patients,” and “provide documentation (eg, update medical records) about patients,” and requested that respondents rate the likelihood of these tasks being replaced by future technology. An additional goal was to compare students’ responses with those in the original UK survey.

Replicating the original survey, the first set of 6 survey items in Section B opened with a brief statement: “Some people believe that machine learning/artificial intelligence will lead to significant changes in medical practice and that machines will one day replace the work of physicians; others deny that new technologies will ever have the capacity to replace this work.” We then asked respondents their opinion on the likelihood that, “future technology will be able to fully replace and not merely aid human doctors in performing each task as well as or better than the average GP.” Employing 4-level Likert items, we included the following response options: “extremely unlikely,” “unlikely,” “likely,” and “extremely likely.” Participants who responded that replacement was “likely” or “very likely” were asked a follow-up question about how soon in their estimation would technology have the capacity to perform the task as well as or better than the average GP, and were provided with a list of 5 response options: “0-4 years from now,” “5-10 years from now,” “11-25 years from now,” “26-50 years from now,” and “more than 50 years from now.” In all closed-ended questions in the survey, we avoided “don’t know,” “neutral,” or “no opinion” options on the grounds that participants often conflate these answers [19].

The study team also extended and developed the original survey instrument by asking students 2 additional questions in Section B. One question was “In 25 years, of the following options, in your opinion what is the likely impact of artificial intelligence/machine learning on the work of GPs?” Students were offered 1 of 4 response options: “no influence (GPs’ jobs will remain unchanged),” “minimal influence (GPs’ jobs will change slightly),” “moderate influence (GPs’ jobs will change substantially),” and “extreme influence (GPs’ jobs will become obsolete).” Participants who answered that there would be minimal, moderate, or extreme influence were then asked the following open comment box question: “Please briefly describe the way(s) in which you believe artificial intelligence/machine learning will change GPs’ jobs in the next 25 years.”

While Section B explored opinions about the potential capabilities of future technology to fully replace GPs on specific tasks, the aim of Section C was to explore students’ views about routine partnership between “man and machine,” that is, GPs and digital tools, in performing various tasks in primary care. Specifically, our aim was to explore students’ predictions about the roles of technology in triage decisions, clinical decision support, remote monitoring of symptoms, and patients’ access to their records. Using a 6-point Likert scale we asked students their level of agreement about the following 6 scenarios: “25 years from now…” (1) “…technology (eg, smartphone apps) will be used to decide when patients need to see a GP,” (2) “…GPs will routinely work in partnership with artificial intelligence/machine learning to diagnose patients,” (3) “…GPs will routinely work in partnership with artificial intelligence/machine learning to determine the likely course of a patient’s illness,” (4) “…GPs will routinely work in partnership with artificial intelligence/machine learning to devise patient treatment plans,” (5) “…remote monitoring of patients’ vital signs will be more common than in-person check-ups of vital signs with GPs,” and (6) “… patients will have greater access to their own medical records than they do today.”

Section D of the survey focused on students’ views about the potential benefits and harms of AI/ML in medicine, and Section E focused on students’ experiences and opinions about formal teaching of AI/ML in their medical degree program. The results of Section D will be published elsewhere, and the results of Section E have now been published [20].

The survey was devised in consultation with Irish, British, and American primary care physicians, and we piloted the survey with physicians in Ireland and the United Kingdom (n=6), and final-year medical students in the United Kingdom (n=5) to ensure face validity. The feedback process was conducted via one-on-one consultations involving think-aloud methods with primary care physicians and medical students.

### Data Analysis

#### Quantitative Component

After survey collection, quantitative survey responses were entered into Excel (Microsoft Corp), and descriptive statistical analysis was carried out using JASP (0.9.2; University of Amsterdam) and SPSS (version 27; IBM Corp). CIs were calculated using the package “REdaS” and function “freqCI,” with the CI level set at 0.95. We used descriptive statistics to examine students’ characteristics and their opinions about the impact of future technology to replace the current tasks of GPs in primary care, whether they believed AI/ML would impact the work of GPs 25 years from now, and whether GPs would routinely partner with AI/ML. For comparisons, students intending to become GPs and internists were grouped together as “planned nonspecialists,” while the remaining categories were grouped together as “planned specialists.” We also embedded into the survey the term “internists” (which is less common in Ireland and the United Kingdom), as we anticipated a high proportion of nonnative student respondents. Due to the ordinal nature of the dependent variables, group comparisons (across males versus females and planned specialists versus planned nonspecialists) were performed using the Mann-Whitney *U* test where the *U* value refers to the difference in the summed ranks.

#### Qualitative Component

Survey responses were uploaded to the software QCAmap (coUnity Software Development GmbH) for analysis. Thematic content analysis was used to investigate students’ responses, and transcripts were read by AK and CB to achieve familiarization with the responses. Owing to limitations with the data set (short phrases or fragments of sentences), full thematic analysis was not applicable [21]. One coder (AK) undertook the thematic analysis. A process was employed in which brief descriptive labels (“codes”) were applied to comments, and multiple codes were applied if comments presented multiple meanings. Following this process, revisions and refinements of codes were undertaken by CB, and AK and CB met to discuss coding decisions. Afterwards, first-order codes (“categories”) were grouped into second-order themes based on commonality of meaning, and AK and CB met to review and refine the final themes.

## Results

### Results of the Quantitative Survey

#### Survey Participants

Data collection at 1 medical school (University College Dublin) was terminated in March 2020 because of teaching disruption due to COVID-19, and survey data from this site was excluded from the analysis. A total of 43.1% (252/585) of final-year students across the 3 remaining medical schools responded (raw data are presented in Multimedia Appendix 2). Among all respondents, 62.6% (157/251) were female and 90.7% (223/246) were born in 1992 or later (Table 1). Participants were nationally diverse, with 57.9% (114/197) from Ireland, 12.2% (24/197) from Malaysia, 12.7% (25/197) from the United Kingdom, and 8.1% (16/197) from Canada. Among the respondents, 69.9% (165/236) identified as White and 27.1% (64/236) identified as Asian. Almost half of all participants (116/247, 47.5%) planned to specialize in general practice or internal medicine (Table 2).

**Table 1:** Participant characteristics.

Characteristic			Value	
**Gender (n=251), n (%)**				
	Female	157 (62.6)		
	Male	94 (37.5)		
Birth year, mean (SD)			1994.3 (2.6)	
**Birth year groups (n=246), n (%)**				
	1980-1984	5 (2.0)		
	1985-1989	9 (3.5)		
	1990-1994	76 (29.7)		
	1995-1999	156 (60.9)		
**Graduate-entry student (n=250), n (%)**				
	Yes	55 (22.0)		
	No	195 (78.0)		
**Nationality^a^ (n=197), n (%)**				
	British/United Kingdom^b^	24 (12.2)		
	Canadian	16 (8.1)		
	Irish	114 (57.9)		
	Malaysia	25 (12.7)		
	Singapore	9 (4.6)		
	Other: Africa	2 (1.0)		
	Other: Asia	6 (3.0)		
	Other: Europe	2 (1.0)		
**Race/ethnicity (n=236), n (%)**				
	Arab	3 (1.2)		
	Asian	64 (27.0)		
	Black	2 (0.9)		
	White	165 (69.9)		
	Multiracial	2 (0.9)		

^a^Nationality categories are not mutually exclusive. In addition, 1 student reported 2 nationalities.

^b^Includes English and Welsh.

**Table 2:** Planned medical specialty.

Planned medical specialty	Value (N=247), n (%)
Anesthetics	13 (5.3)
Dermatology	2 (0.8)
Elderly care or geriatrics	2 (0.8)
Emergency medical services	3 (1.2)
General practice/internal medicine	116 (47.5)
General surgery	19 (7.8)
Ophthalmology	3 (1.2)
Other surgery specialty	31 (12.7)
Obstetrics & gynecology	7 (2.8)
Pediatrics	20 (8.2)
Pathology (any subspecialty)	3 (1.2)
Psychiatry	7 (2.8)
Radiology	2 (0.8)
Other	8 (3.2)
Do not know/unsure	11 (4.4)

#### Work of GPs in the Long Term: Opinions About Technological Replacement

Around two-thirds of participants (158/251, 62.9%) reported it was “very unlikely” or “unlikely” that technology would ever be able to fully replace GPs in reaching diagnoses (Table 3). Among the remaining 37.1% (93/251) who thought it was “likely” or “very likely,” only 22% (20/93) estimated that the capacity for replacement would emerge in 0-10 years, with many (38/93, 41%) estimating a time scale of 11-25 years (Table 4). Similarly, most participants (157/245, 64.1%) reported it was “very unlikely” or “unlikely” that future technology would be able to fully replace GPs in formulating personalized treatment plans. Among those who believed this was likely or very likely, however, 41% (36/87) estimated that the technological capacity to do so would emerge in 11-25 years.

**Table 3:** Opinions about the likelihood of future technology replacing general practitioner tasks.

Task	Opinion									
	Very unlikely		Unlikely			Likely			Very likely	
	Value, n (%)	95% CI^a^	Value, n (%)	95% CI^a^	Value, n (%)		95% CI^a^	Value, n (%)		95% CI^a^
1. Analyze patient information to reach diagnoses (N=251)	40 (15.9)	11.4-20.5	118 (47.0)	40.8-53.2	75 (29.9)		24.2-35.5	18 (7.2)		4.0-10.4
2. Analyze patient information to predict the likely course of the patient’s illness (N=248)	20 (8.1)	4.7-11.5	92 (37.1)	31.1-43.1	116 (46.8)		40.6-53.0	20 (8.1)		4.7-11.5
3. Evaluate when to refer patients to other health professionals (N=246)	26 (10.6)	6.7-14.4	100 (40.7)	34.5-46.8	101 (41.1)		34.9-47.2	19 (7.7)		4.4-11.1
4. Formulate personalized treatment plans for patients (N=245)	45 (18.4)	13.5-23.2	112 (45.7)	39.5-52.0	71 (29.0)		23.3-34.7	17 (6.9)		3.8-10.1
5. Provide empathetic care to patients (N=247)	182 (73.7)	68.2-79.2	49 (19.8)	14.9-24.8	15 (6.1)		3.1-9.1	1 (0.4)		0.0-1.2
6. Provide documentation (eg, update medical records) about patients (N=247)	7 (2.8)	0.8-4.9	28 (11.3)	7.4-15.3	118 (47.8)		41.5-54.0	94 (38.1)		32.0-44.1

^a^Lower bound CIs have been set to 0.

**Table 4:** Opinions about time scale for technological capacity to emerge.

Task	Time scale^a^													
	0-4 years			5-10 years			11-25 years			26-50 years			>50 years	
	Value, n (%)	95% CI^b^	Value, n (%)		95% CI^b^	Value, n (%)		95% CI^b^	Value, n (%)		95% CI^b^	Value, n (%)		95% CI^b^
1. Analyze patient information to reach diagnoses (N=93)	2 (2.2)	0.0-5.1	19 (20.4)		12.2-28.6	38 (40.9)		30.9-50.9	25 (26.9)		17.9-35.9	9 (9.7)		3.7-15.7
2. Analyze patient information to predict the likely course of the patient’s illness (N=138)	5 (3.6)	0.5-6.7	24 (17.4)		11.1-23.7	53 (38.4)		30.3-46.5	36 (26.1)		18.8-33.4	20 (14.5)		8.6-20.4
3. Evaluate when to refer patients to other health professionals (N=121)	7 (5.8)	1.6-9.9	34 (28.1)		20.1-36.1	48 (39.7)		31.0-48.4	26 (21.5)		14.2-28.8	6 (5.0)		1.1-8.8
4. Formulate personalized treatment plans for patients (N=87)	6 (6.9)	1.6-12.2	24 (27.6)		18.2-37.0	36 (41.4)		31.0-51.7	12 (13.8)		6.6-21.0	9 (10.3)		4.0-16.7
5. Provide empathetic care to patients (N=18)	0 (0)	N/A^c^	3 (16.7)		0.0-33.9	3 (16.7)		0.0-33.9	8 (44.4)		21.5-67.4	4 (22.2)		3.0-41.4
6. Provide documentation (eg, update medical records) about patients (N=214)	52 (24.3)	18.6-30.1	86 (40.2)		33.6-46.8	50 (23.4)		17.7-29.0	20 (9.3)		5.5-13.3	6 (2.8)		0.6-5.0

^a^Participants were only asked to indicate time scale if they first indicated it was likely or very likely that future technology will fully replace human doctors in each task as well as or better than the average general practitioner. As such, some data are not provided (missing n=159, 138, 121, 165, 234, and 38 for tasks 1, 2, 3, 4, 5, and 6, respectively).

^b^Lower bound CIs have been set to 0.

^c^N/A: not applicable.

Participants were divided about the technological capacity to fully replace GPs regarding prognoses or referrals. For prognoses and referrals, 54.9% (136/248) and 48.8% (120/246), respectively, indicated replacement was “likely” or “very likely,” and a majority of these participants believed that the timeframe for this capacity for prognoses and referrals was 11-25 years (53/128, 38.4% and 48/121, 39.7%, respectively). In contrast, 85.9% (212/247) predicted technology would be able to fully replace GPs in undertaking documentation, and among them, 64.5% (138/214) predicted this capacity would emerge within 10 years. Finally, participants were least expectant about the potential for technology to replace GPs in providing empathetic care, with 93.5% (231/247) predicting this was “very unlikely” or “unlikely.”

#### Work of GPs in 25 Years: Opinions About the Impact of AI/ML

**Figure 1:**
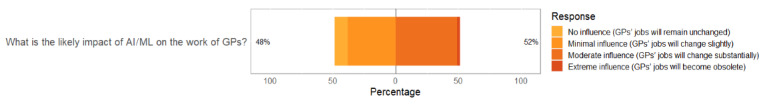
Predicted impact of artificial intelligence/machine learning on the work of general practitioners in the next 25 years. AI: artificial intelligence; GP: general practitioner; ML: machine learning.

Around half of the surveyed students (127/246, 51.6%) believed AI/ML would have a moderate or extreme influence on the work of GPs in the next 25 years (Figure 1). Around 1 in 10 (25/246, 10.2%) believed it would have no influence, with the work of GPs remaining unchanged.

**Figure 2:**
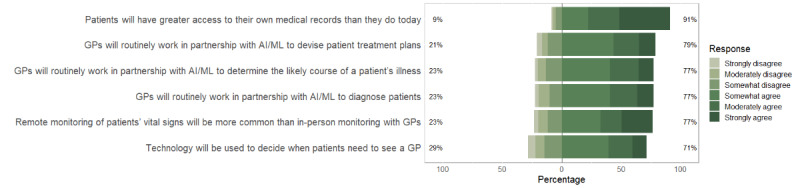
Predicted impact of artificial intelligence/machine learning on health care in the next 25 years. AI: artificial intelligence; GP: general practitioner; ML: machine learning.

When asked to reflect on what, specifically, might change 25 years from now, around one-third “moderately” or “strongly” agreed that technology (eg, smartphone apps) would be used to decide when patients need to see a GP (79/244, 32.2%), with similar proportions predicting GPs would routinely work in partnership with AI/ML to diagnose patients (90/244, 36.9%), determine the likely course of a patient’s illness (90/244, 36.9%), or devise patient treatment plans (86/244, 35.2%) (Figure 2). More than 4 in 10 (107/244, 43.9%) “moderately” or “strongly” agreed that in 25 years from now, remote monitoring of patients’ vital signs will be more common than in-person check-ups of vital signs, with the majority (169/244, 69.3%) “moderately” or “strongly” agreeing patients will have greater access to their own medical records than they do today.

#### Correlates of Opinions

Male students in our sample rated it more likely that future technology would fully replace GPs in undertaking diagnoses (Mann-Whitney *U*=6137.5; *P*=.02), prognoses (*U*=5254; *P*<.001), and empathy (*U*=6108; *P*=.02), compared with female students. No other gender differences were observed in participants’ forecasts. The likelihood of future technology replacing GPs for referrals was rated higher by students who planned to specialize in medical professions other than general practice or internal medicine (“planned specialists”) than by those who planned to enter primary care professions (Mann-Whitney *U*=5501; *P*<.001). Similarly, making forecasts about the impact of technology on the work of GPs 25 years from now, planned specialists thought that AI/ML would have a large impact (*U*=5972.5; *P*=.02), more strongly agreed that technology would be routinely used to decide when patients need to see a GP (*U*=5343; *P*=.001), and agreed that GPs would routinely work in partnership with AI/ML to diagnose patients (*U*=5445; *P*=.003) and determine the likely course of a patient’s illness (*U*=5207; *P*<.001). Finally, compared with aspirant nonspecialists, planned specialists more strongly predicted that 25 years from now, patients will have greater access to their own medical records (*U*=5656.5; *P*=.01).

### Results of the Qualitative Survey

In total, 60.7% (153/252) of students left comments describing the ways in which they believed AI/ML will change the work of GPs 25 years from now. Comments were short and had a mean of 7.21 (SD 6.96) words. Following inductive analysis (see above), 4 major themes emerged (see Textbox 1). Illustrative examples of themes and categories are provided below. For more elaborate comments, participant number, gender, year of birth, and chosen medical specialty have been mentioned (the latter information was provided by the respondents).

**Themes and categories.**

**Administrative effects**

- Reduction/removal of administrative tasks and workload
- “Better administration culture”
- Greater efficiency of care
- Improved communication within health care
- Artificial intelligence/machine learning (AI/ML) will assist with documentation
- Increased use and/or quality of patient health records

**Clinical judgement**

AI/ML will …

- Triage
- Assist in diagnosis
- “There will be less expected of general practitioners (GPs)”
- Replace GPs
- Not replace GPs
- Assist decision-making
- Decrease error rate

**Care management and access**

AI/ML will …

- Assist in treatment and/or management
- Enable patient self-monitoring
- Increase telemedicine
- Monitor/analyze disease progression
- Assist medication prescribing
- Make some technologies more accessible
- Gather data outside consultation
- Introduce financial challenges

**Relational aspects**

AI/ML will …

- Increase time with patients
- Reduce time with patients
- Better human interaction
- Not replace empathy
- Not impact patient-doctor relationship
- Introduce ethical issues
- Not replace human interaction
- Impact patient-doctor relationship

#### Administrative Effects

Students envisaged changes to administrative work because of AI/ML as having the biggest effect on the work of GPs, with 33.9% (108/319) of all coded passages belonging to this theme. Two-thirds of the coded phrases within the theme described a reduction or complete removal of administrative tasks and workload (40/108, 37.0%), or assistance with documentation (31/108, 28.7%). Students frequently forecasted “less paperwork” and “easier paperwork” as likely, with some comments suggesting AI/ML would reduce the time needed to process documents requested by patients (eg, “sick letters” or “referral letters”). When predicting how technological advancements would assist with documentation, few students elaborated beyond describing it as “better” or “easier.” Some examples referred to automation with respect to notetaking (“automatic dictation instead of typed/written notes” [Participant #165, female, born 1997, internal medicine specialty]) both for referrals and appointment summaries.

Greater efficiency in care (22/108, 20.4%) was also a common category within this theme. Students described several ways in which they believed AI/ML “may streamline care” [Participant #242, male, born 1993, psychiatry specialty], for example, by optimizing resource allocation or the referral infrastructure:

Better links between primary and secondary care - information from both will be better shared.

Participant #230, female, born 1995, anesthetics specialty

Students also expected better time management as a result of automation of simple tasks like filing referrals or reviewing basic information from examinations, for example, “Gathering and collating data will become easier and assist GPs in their work.” [Participant #195, male, born 1996, general practice specialty].

Improved use of patient health records was another category, and students predicted AI/ML would provide easier access to the records both among GPs and other specialists, as well as assist in populating the records (“updating records and summarizing consultation” [Participant #127]).

#### Clinical Judgment

A second major theme was clinical judgment (94/319, 29.5% of all coded passages), which encompassed predictions about how GPs’ clinical decision-making may be affected by AI/ML. Assistance in diagnosis was a major concern among students, with half (47/94, 50%) of the coded passages in this theme describing various AI/ML applications. Students envisaged AI algorithms that will provide “diagnosis based on symptom consultation” [Participant #93, female, born 1996, general practice specialty], particularly when it comes to dermatology, hematology, radiology, and other medical imaging. Some described a degree of sophistication:

data interpretation according to data banks may play a larger role

Participant #207, female, born 1995, internal medicine specialty

providing differential diagnoses

Participant #56, female, born 1996, other surgery specialty

Others were more reserved and were more doubtful about the impact of AI/ML:

In terms of diagnosis, medicine is as much an art as a science. I find it difficult to believe that a computer can appreciate the value of a clinical decision based on observation and relationships with a patient.

Participant #215, female, born 1995, internal medicine specialty

Predictions about the effects of AI/ML tools on decision-making (10/94, 11%) and triage (15/94, 16%) were also common, and forecasts included the idea that AI/ML might serve as support tools for GPs by “reducing waiting lists,” “helping screen patients,” or “reviewing appointments.” Some suggested that in 25 years, “there will be less expected of GPs” [Participant #19, male, born 1990, emergency medicine specialty], with some disagreement on the scope of AI/ML to replace them altogether. There were concerns that “GPs could be entirely supplanted by artificial technology in 25 years” [Participant #167, male, born 1996, internal medicine specialty], which may lead to a “lack of jobs” [Participant #163, male, born 1995, obstetrics specialty]. One student perceived developments in AI/ML as a threat to the GP profession only:

In terms of providing empathy or communicating directly with patients, nurse practitioners are already a less expensive and equally as knowledgeable alternative to GPs that could work in tandem with AI to render the GP entirely obsolete.

Participant #167, male, born 1996, internal medicine specialty

A few believed that technology will, in the words of 1 participant, “function as an adjunct rather than replacement” [Participant #166, female, born 1997, anesthetics specialty].

#### Care Management and Access

Another theme was care management and access (94/319, 29.5% of all coded passages). Within it, the leading prediction was that AI/ML would aid in treatment or management (34/94, 36%) of care, including “formulating treatment plans” and “referral pathway suggestions.” Some were cautious, limiting their predictions to “*simple* conditions, eg, common cold” [Participant #162, female, born 1996, psychiatry specialty], while others saw significant potential. One student mentioned that AI/ML “could help organize patients’ treatment regime based on multiple factors such as compliance” [Participant #186, female, born 1995, general surgery specialty]. Medication prescribing was also perceived as likely to be impacted by AI/ML (29/94, 31%). From automatic prescribing and renewal to contraindication analysis and error detection, students commonly forecasted a role for AI/ML in medication management. Several commenters predicted timely personalized prescribing based on “test results,” “guidelines,” and “adverse effects reviews.”

Predictions about approaches to treatments enabled by technology were further reflected in the category monitoring/analysis of disease progression (10/94, 11%). Patient “disease course prediction” was expected to be supplemented through “vitals and timeline analysis” enabled by AI/ML advances. Remote health care tools were also referenced (5/94, 5%) via “pre-examination before consultation” [Participant #23, female, born 1994, general medicine specialty] and patient self-monitoring (3/94, 3%). Only a few comments (7/94, 8%) discussed telemedicine, forecasting “less in-person visits” [Participant #171, female, born 1997, pediatrics specialty] and “video consultations” [Participant #229, female, born 1991, general medicine specialty]. Similarly, a minority (4/94, 4%) considered that the implementation of AI/ML would make care more accessible 25 years from now, though some believed it would also introduce financial challenges.

#### Relational Aspects

The smallest emergent theme encompassed the impact of AI/ML on relational aspects of care (23/319, 7.2% of all coded passages), which focused on opinions about how technology might change the patient-GP relationship. Within this theme, students were divided about whether technological advancements might increase (3/23, 13%) or decrease (4/23, 17%) time spent with patients. Students, however, were skeptical about the replacement of human interactions by AI/ML within 25 years, particularly regarding empathy provided by GPs:

Machines will perform logical work whereas GPs would manage the humanity side of the medical work, ie, empathy, support, encouragement.

Participant #154, male, born 1995, hospital management specialty

Students described the patient-doctor relationship as follows: “key importance for patients’ benefit and it is therapeutic” [Participant #114, female, born 1993, unsure about specialty], with only 2 (9%) respondents predicting it could be enhanced through advances in AI/ML. Only 1 person (4%) predicted a negative relational effect of AI/ML stating “poor rapport” [Participant #189, male, born 1997, internal medicine specialty]. A similar minority (4/23, 17%) of codes pertained to the ethical implications of adopting AI/ML in health care. Only 1 (4%) participant described concerns about patients’ privacy as a consequence of AI/ML innovations.

## Discussion

### Summary of the Major Findings

Few studies have explored the views of medical students about how AI/ML will impact the future of their job. This mixed methods study specifically explored forecasts of final-year Irish medical students about how future technology might influence the work of GPs. When requested to forecast the impact of AI/ML on the work of GPs 25 years from now, students were divided, with around half of all surveyed students believing the work of GPs will change minimally or not at all. Notably, students who did not intend to enter primary care predicted that AI/ML would have greater impact.

With regard to specific tasks, around one-third of students moderately or strongly agreed that 25 years from now, technology (eg, smartphone apps) would be used to decide when patients need to see a GP. Similarly, around one-third moderately or strongly agreed that GPs would routinely work in partnership with AI/ML to diagnose patients, determine the likely course of a patient’s illness (“prognosis”), or devise patient treatment plans. About 4 in 10 students moderately or strongly agreed that 25 years from now, remote monitoring of patients’ vital signs would be more common than in-person check-ups for vital signs, with 7 in 10 students agreeing that patients would have greater access to their medical records. Again, students who did not intend to enter primary care were more likely to forecast that AI/ML would impact key aspects of the work of GPs, including formation of decisions about when patients should see GPs, assisting GPs in diagnoses and prognoses, and helping patients obtain greater access to their medical records.

Results from the qualitative section of the survey supported and partially elaborated on these predictions. The dominant perspective was that 25 years from now, there would be a reduction in GPs’ workloads with less paperwork and greater efficiency in primary care. Other common themes encompassed forecasts that AI/ML-enabled tools would aid clinical judgment but only for a narrow range of symptoms, mostly pertaining to imagery. Another theme was the potential for AI/ML to aid with treatment and care management, including automatic prescribing. Fewer students envisaged a role for AI/ML in patient self-monitoring, and only a minority predicted an increase in telemedicine or patient access to health care. Although participants were divided about whether AI/ML might have an impact on the time GPs would spend with patients, most were skeptical about whether technological tools could ever replace the empathy provided by GPs.

Offering forecasts on the capacity for future technology to fully replace core aspects of the job, around 2 in 3 students believed it is unlikely or very unlikely that GPs would ever be fully replaced by AI/ML tools in performing diagnoses or formulating personalized treatment plans for patients. Students were split over whether prognoses or triage could ever be fully replaced. Consistent with the qualitative component, however, students were most skeptical about the scope of future technology to replace GPs in providing empathic care, with more than 9 in 10 predicting this was unlikely or very unlikely. In contrast and in keeping with predictions about the impact of technology over the next 25 years, students were most expectant about the scope of future technology to fully replace GPs in undertaking the role of documentation, with more than 8 in 10 believing this was likely or very likely. Among them, around 2 in 3 predicted this would happen in the next 10 years. Finally, we also found correlations between gender and students’ opinions, with male respondents more likely to believe future technology would fully replace GPs in undertaking diagnostics and prognostics, and in the provision of empathy. Students who did not intend to enter primary care professions were more likely to believe GPs would be replaced by future technology in making referral decisions to other specialists.

The results of this study mirror other recent medical student and GP surveys, which demonstrated a wide range of opinions among participants about the impact of AI/ML on health professions [14,17,18]. Conspicuously, students’ opinions about the prospects for technology to fully replace various primary care tasks revealed some similarities but also intriguing differences with the findings in a recent survey conducted with GPs in the United Kingdom [9]. Final-year medical students in Ireland and experienced GPs offered similar predictions about the capacity for future technology to replace GPs in key tasks; however, students tended to be more cautious and conservative in their estimations of time scales for when AI/ML advances might arise. Although these divergences might be associated with sampling effects, we noted that the original UK GP study [9] reported a weak correlation between respondent age and opinions, with younger GPs more skeptical about the imminence of technological advances.

The reasons behind associations between younger age/inexperience and relative technoskepticism are not fully understood, though 2 hypotheses might be considered. First, it is reasonable to hypothesize that, compared with established GPs, younger respondents may be more AI/ML savvy and less susceptible to hype about AI/ML, and as a result, they may be more reserved in their forecasts. However, a growing number of student surveys now indicate that there is scarce formal training in AI/ML in medical schools [12-18,20]. Indeed, in previously published findings that emerged from the present survey, 4 in 10 final-year students reported that they had not heard of the term “machine learning,” with 2 in 3 reporting spending no time learning about AI/ML during the entire period of their medical degree [20]. Therefore, it is unlikely that greater awareness about technology influenced comparatively more conservative predictions among our student respondents. A second and more plausible hypothesis is that younger age/inexperience and technoskepticism might be associated with well-documented optimism bias, which is the tendency of people to believe that they will not be affected by negative events. In short, student participants may be susceptible to interpreting information on AI/ML in ways that support the prospects of their own long-term career in medicine. Tentative support for this hypothesis comes from differences in opinions related to students’ choices of medical specialty, with those intending to enter primary care less likely to believe AI/ML would impact the work of GPs. Further support comes from the finding that students predicted that the administrative burdens of updating documentation would be outsourced to technology.

Like other studies, including those among psychiatrists [10], male respondents were more likely to predict that future technology will be able to fully replace GPs in some key tasks. The reasons for this difference are not fully understood, though findings from social psychology demonstrate sex differences when it comes to risk aversion [22]. It may be that males are slightly less cautious on average compared with females in offering professionally threatening predictions. It is worth emphasizing, however, that other surveys have not reported gender differences [9]. Only one-third of our respondents were male. For many years in Ireland, there has been a trend of more male medical students than female medical students [23]. Therefore, it is possible that gender disparities in respondents’ opinions in the present study might have been an artifact of sampling limitations.

We also observed contrastive predictions among our students compared with informaticians and other experts working in health care AI/ML and related fields. A Delphi poll of international health informatics experts reported consensus that in 10 years (by 2029), advances in AI/ML would prompt workforce changes within primary care, with a shift toward computing and engineering in the educational backgrounds of students entering medical school, and increasing demands on students to work with AI/ML-enabled tools in health care [24]. In contrast, when asked to reflect on what might change 25 years from now, a minority of students forecasted that GPs would partner with AI/ML tools in supporting clinical decision-making. However, such advances are already underway. In countries with electronic health records (EHRs) in primary care, the availability and uptake of clinical decision support systems, which are tools that link patients’ personal information held in EHRs to clinical software to inform patient-specific assessments or recommendations, appear to be widespread [25]. These tools are being increasingly powered by ML, and they use computerized reminders, alerts, and prompts linked to patients’ electronic records to help inform recommendations. Prescription alerts, for example, warn doctors about harmful dosing or risks of drug interactions, and clinical decision support systems have the potential to help standardize guideline adherence, and support diagnostic and prognostic decisions [26].

Other predictions associated with access to primary care and patient management of their care also diverged from expert predictions and current trends. For example, when asked to predict what might change 25 years from now, a minority of students agreed that technology, such as smartphone apps, would be routinely used to decide when patients need to see a GP, a finding supported by qualitative analysis. Although partly accelerated by COVID-19 and stay-at-home measures, so-called “digital first” gateways to online triage, such as AskMyGP, Engage Consult, and eConsult in the United Kingdom, are being increasingly adopted in primary care [27]. Although these systems are implemented with the goal of mitigating increased work burdens, it is important to note that there is scarce evidence such systems, as currently embedded into work practices, do in fact improve efficiencies, and they may even exacerbate pressures on physicians by identifying greater patient needs [28,29]. It is worth emphasizing, however, that predictions about increasing implementation of AI/ML tools in medicine are not the same as gauging views about their adequacy, safety, or ease of use, especially with respect to integration into GPs’ workflows. Notwithstanding, students’ predictions did appear to contrast with growing prepandemic secular trends.

A larger proportion (107/244, 43.9%) of students, though still a minority, moderately or strongly agreed that remote monitoring of vital signs will be more common than in-person check-ups in the near future. Nonetheless, few students elaborated on this in the qualitative section of the survey. Although students could not have predicted how the pandemic would catalyze an uptick in telemedicine, including the use of electronic communication to track, monitor, or manage symptoms or chronic conditions [30], interest and uptake in remote patient monitoring has grown in recent years [31,32]. Increasingly via smartphone photos, blood pressure cuffs, heart rate monitors, portable electrocardiography systems, and a host of other devices, patients can manage their health from their home with real-time readings relayed instantly to the patients and the clinical team. Moreover, there is evidence that so-called mobile health may improve precision [33-36] while driving down health care expenditure, including hidden travel costs, related to in-person appointments [37-39].

Finally, 1 prediction was fully in line with recent health care developments. Almost all students expected that access to medical records would increase in the next 25 years. Currently, in around 20 countries, including Australia, Canada, the Nordic countries, and the United States, patients are offered rapid online access to at least some of their EHRs, a practice that is growing.

### Strengths and Limitations

A major strength was soliciting the opinions of a diverse range of medical students from institutions in geographically distinctive regions of Ireland. The survey offered insights into students’ forecasts about the potential impact of technology on the work of GPs both in the medium term during their own career span and in the longer term with regard to replacement of doctors. Going further than other investigations [9], the present study examined students’ views about the likelihood of full technological replacement of GPs in specific core roles while also examining participants’ predictions about the extent to which GPs might partner with machines in a variety of tasks. Combined with the mixed methods approach, the study permitted more nuanced students’ opinions about the impact of AI/ML on the work of GPs.

The survey has several limitations. We used a nonprobability convenience sample, limiting generalizations about the opinions of all final-year medical students in Ireland. In addition, the moderate response rate (43%) raises questions about representativeness, though this is a very strong response rate for survey research where participants do not receive compensation. It is also unknown whether the decision to complete the survey was influenced by prior awareness about AI/ML or whether response biases were influenced by participants who were more enthusiastic or more skeptical about the effects of AI/ML on primary care. Because of the limitations of open comment boxes, participants’ responses were often vague or truncated, and it was not possible to probe the views of students in depth. The survey was administered prior to the COVID-19 pandemic, which has been associated with considerable developments and attention regarding the role of AI/ML-enabled tools in digital epidemiology and public health. Conceivably, if the survey had been undertaken after the pandemic, participants’ views might have differed. Nonetheless, to date, no medical school included in this survey has modified their curriculum to include greater education about AI/ML.

Further survey research and curricular analyses could explore the extent to which medical students receive training about existing clinical decision support tools and their implementation in clinical work. Qualitative research methods, such as interviews and focus groups, could provide more nuanced findings on aspects of students’ views and understanding about the impact of AI/ML on medicine. Finally, future studies could explore the views of medical faculty about the impact of AI/ML-enabled tools on medicine, including their awareness, understanding, and appreciation of the scope for these applications and limitations associated with these applications. Such work might help illuminate potential obstacles to curricular advancement on these topics within medical education.

### Conclusions

This mixed methods survey provides insights into what final-year medical students in Ireland think about the impact of AI/ML on primary care. A broad spread of opinions was apparent, with many forecasts contrasting with the considered opinions of health informaticists. Ireland is ranked as a leading technology capital in Europe [40], with the fastest growing technology workforce on the continent [41]. This survey combined with previously published findings [20] suggests that training regarding AI/ML in Irish medical education may be lagging behind advances in the field. We caution that without a firm curricular foundation on advances in AI/ML, students may rely on extreme perspectives involving self-preserving optimism biases that demote the impact of advances in technology on their choice of specialty on the one hand and technohype on the other. Ultimately, these biases may lead to negative consequences in health care. Improvements in medical education could help prepare tomorrow’s doctors to optimize and lead the ethical and evidence-based implementation of AI/ML-enabled tools in medicine for enhancing the care of tomorrow’s patients.

## Appendix

Multimedia Appendix 1 Medical school student survey.

Multimedia Appendix 2 Raw study data.

## Abbreviations

AI: artificial intelligence
EHR: electronic health record
GP: general practitioner
ML: machine learning
