# Supplementary material for: Computerization of the Work of General Practitioners: Mixed Methods Survey of Final-Year Medical Students in Ireland
Source: JMIR Med Educ. 2023 Mar 20;9:e42639. doi: 10.2196/42639 (PMC10131917; doi:10.2196/42639)
Supplement: Multimedia Appendix 1 [file mededu_v9i1e42639_app1.docx]

**Multimedia Appendix 1.** Medical school student survey.

**MACHINE LEARNING AND MEDICINE:**

**OPINIONS OF STUDENTS IN IRISH MEDICAL SCHOOLS**

**Information Sheet and Consent Form**

Thank you for considering participating in this research project. The purpose of this document is to explain to you what the work is about and what your participation would involve, so as to enable you to make an informed choice.

The purpose of this study is to investigate the opinions of final year medical students in Ireland about technology and the future of medicine. We do not expect any knowledge of technologies: instead we are interested in your views as students’. Should you choose to participate, you will be asked some non-identifying demographic information, followed by questions with multiple choice answers asking you to express your level of agreement with various statements about how medicine might be impacted by technology. We are also interested in any comments you might have, should you feel able to provide them. The survey will take around 10-15 minutes to complete.

Your responses will help us to better understand the opinions of future medical professions on artificial intelligence, which in turn may help to inform medical curricula. While there are no direct benefits to you, participating in this survey may help to stimulate you to think about the role of technology in healthcare.

Participation in this study is completely voluntary. There is no obligation to participate, and should you choose to do so you can refuse to answer specific questions, or decide to withdraw from the study. We will not collect any personal or sensitive information therefore the survey will be fully anonymous. The survey is not a test, and your decision to participate will not affect your grades. Ticking the box below will indicate consent to participate.

You maintain the right to withdraw from the study at any stage up to the point of data submission. At this point your data will be collated with that of other participants and can no longer be retracted.

The anonymous data will be stored securely for up to ten years on the NUIG Server in secured form. The information you provide may contribute to research publications and/or conference presentations which may be publicly available. However, your contributions will be fully anonymous.

We do not anticipate any negative outcomes from participating in this study.

This study has obtained ethical approval from the Research Ethics Committee at NUIG.

If you have any queries about this research, you can contact Dr Muiris Houston at [muiris.houston@gmail.com](about:blank)

If you agree to take part in this study, please complete the consent question below.

**Consent Form**

Do you consent to participate in this study?

Yes ☐

No ☐

**
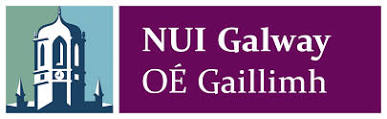
**

**MACHINE LEARNING AND MEDICINE:**

**OPINIONS OF STUDENTS IN IRISH MEDICAL SCHOOLS**

**Dear Student,**

**Researchers at the School of Medicine, NUIG invite you to take part in a survey. We are inviting you, as medical students to give your opinions about technology and the future of medicine. This research will help to inform medical educationalists and policy-makers.**

The survey should take around 10 (and no more than 15) minutes to complete. We will not collect any identifying information from you, and your responses will be fully anonymous. Your response will be collated with those of other respondents in aggregated, anonymous form*.* ***This survey is not a test; we are interested in your opinions as medical students.*** We refer you to the Information and Consent Sheet for more information.

If you decide to participate, we appreciate your time and contribution to our research.

Thank you.

Dr Muiris Houston, NUIG

Email contact: muiris.houston@gmail.com

**SECTION A**

**In this section we will ask demographic questions.**

**A1. Gender. Please select box.**

Male
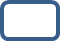
 _1_

Female
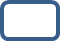
 _2_

Not listed above (please specify)
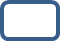
 _3_

Prefer not to answer
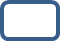
 _4_

**A2. Year of birth. Please enter year.**

**A3. Current year of your medical school education. Please select box.**

1^st
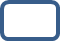
 1^

2^nd
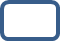
 2^

3^rd
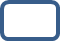
 3^

4^th
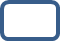
 4^

5^th^  ^
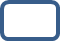
 5^

**A4. (i) Are you a graduate-entry student (‘GEM’)? Please select box.**

No
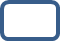
 ^1^

Yes
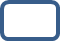
 _2_

1. **If you answered ‘Yes’ what was your undergraduate degree subject(s)? Please enter.**

**A5. What general area of medical specialty do you plan to enter? Please select one box only.**

General practice
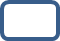
 _1_

Internal medicine (any sub-specialty)
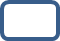
 _2_

Paediatrics
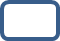
 _3_

Elderly care/ Geriatrics
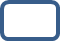
 _4_

General Surgery
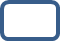
 _5_

Other surgery specialty
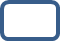
 _6_

Anesthetics
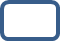
 _7_

Radiology
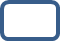
 _8_

Pathology (any sub-specialty)
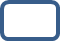
 _9_

Other (please specify)
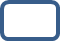
 _10_

**A6. **[*OPTIONAL*] Please enter your nationality.**

**A7. **[*OPTIONAL*] What is your race/ethnicity? Please select.**

**Asian**
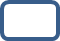
 _1_

**Black**
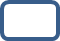
 _2_

**White**
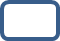
 _3_

**Mixed**
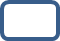
 _4_

**Other** - Please specify
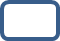
 _5_

**SECTION B**

**The questions in this section are on your opinions about the impact of machine learning/artificial intelligence on the future of primary care.**

**We do not assume you have any knowledge about machine learning or artificial intelligence in medicine.**

**Some people believe that machine learning/artificial intelligence will lead to significant changes in medical practice and that machines will one day replace the work of physicians; others deny that new technologies will ever have the capacity to replace this work.**

**While there is much debate about the impact of machine learning/artificial intelligence on medicine, we are interested in your opinions.**

**In this section we invite you to give your views about whether future technology will be able to perform various medical tasks as well as or better than the average GP.**

**In the questions below, we are interested in whether you believe technology will be able to FULLY REPLACE – NOT MERELY AID – GPs in performing these tasks.**

**The questions begin on the next page.**

**B1. In your opinion, what is the likelihood that future technology will be able to fully replace human doctors to perform the following task as well as or better than the average GP…**

**B1(i) Analyse patient information to reach diagnoses.**

Very unlikely
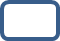
 _1_ Go to **B2**

Unlikely
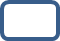
 _2_

Likely
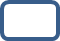
 _3_ First go to **B1(ii)**

Very likely
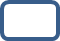
 _4_

**B1(ii) *If you answered Likely or Very likely:***

**When, in your estimation, will future technology have the capacity to fully replace the average GP in performing this task?**

0-4 years from now
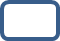
 _1_

5 to 10 years from now
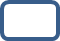
 _2_

11 to 25 years from now
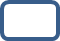
 _3_

26-50 years from now
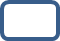
 _4_

More than 50 years from now
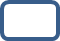
 _5_

**B2.** **In your opinion, what is the likelihood that future technology will be able to fully replace human doctors to perform the following task as well as or better than the average GP…**

**B2(i) Analyse patient information to predict the likely course of the patient’s illness.**

Very unlikely
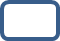
 _1_ Go to **B3**

Unlikely
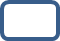
 _2_

Likely
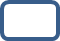
 _3_ First go to **B2(ii)**

Very likely
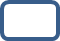
 _4_

**B2(ii) *If you answered Somewhat likely, Likely or Very likely:***

**When, in your estimation, will future technology have the capacity to fully replace the average GP in performing this task?**

0-4 years from now
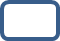
 _1_

5 to 10 years from now
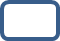
 _2_

11 to 25 years from now
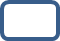
 _3_

26-50 years from now
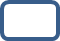
 _4_

More than 50 years from now
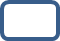
 _5_

**B3. In your opinion, what is the likelihood that future technology will be able to fully replace human doctors to perform the following task as well as or better than the average GP…**

**B3(i) Evaluate when to refer patients to other health professionals.**

Very unlikely
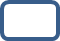
 _1_ Go to **B4**

Unlikely
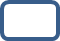
 _2_

Likely
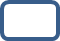
 _3_ First go to **B3(ii)**

Very likely
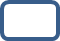
 _4_

**B3(ii) *If you answered Somewhat likely, Likely or Very likely:***

**When, in your estimation, will future technology have the capacity to replace the average GP in performing this task?**

0-4 years from now
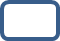
 _1_

5 to 10 years from now
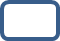
 _2_

11 to 25 years from now
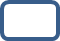
 _3_

26-50 years from now
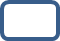
 _4_

More than 50 years from now
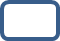
 _5_

**B4. In your opinion, what is the likelihood that future technology will be able to fully replace human doctors to perform the following task as well as or better than the average GP…**

**B4(i) Formulate personalised treatment plans for patients.**

Very unlikely
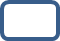
 _1_ Go to **B5**

Unlikely
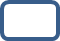
 _2_

Likely
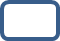
 _3_ First go to **B4(ii)**

Very likely
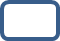
 _4_

**B4(ii) *If you answered Somewhat likely, Likely or Very likely:***

**When, in your estimation, will future technology have the capacity to replace the average GP in performing this task?**

0-4 years from now
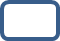
 _1_

5 to 10 years from now
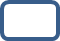
 _2_

11 to 25 years from now
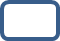
 _3_

26-50 years from now
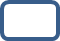
 _4_

More than 50 years from now
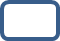
 _5_

**B5 In your opinion, what is the likelihood that future technology will be able to fully replace human doctors to perform the following task as well as or better than the average GP…**

1. **Provide empathetic care to patients.**

Very unlikely
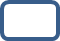
 _1_ Go to **B6**

Unlikely
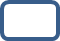
 _2_

Likely
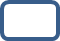
 _3_ First go to **B5(ii)**

Very likely
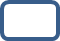
 _4_

**B5(ii) *If answered Somewhat likely, Likely or Very likely:***

**When, in your estimation, will future technology have the capacity to fully replace the average GP in performing this task?**

0-4 years from now
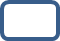
 _1_

5 to 10 years from now
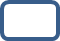
 _2_

11 to 25 years from now
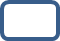
 _3_

26-50 years from now
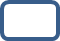
 _4_

More than 50 years from now
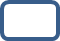
 _5_

**B6. In your opinion, what is the likelihood that future technology will be able to fully replace human doctors to perform the following task as well as or better than the average GP…**

1. **Provide documentation (e.g., update medical records) about patients.**

Very unlikely
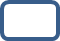
 _1_ Go to **B7**

Unlikely
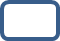
 _2_

Likely
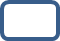
 _3_ First go to **B6(ii)**

Very likely
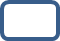
 _4_

**B6(ii) *If answered Somewhat likely, Likely or Very likely:***

**When, in your estimation, will future technology have the capacity to fully replace the average GP in performing this task?**

0-4 years from now
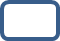
 _1_

5 to 10 years from now
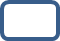
 _2_

11 to 25 years from now
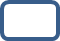
 _3_

26-50 years from now
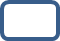
 _4_

More than 50 years from now
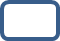
 _5_

**B7. In 25 years, of the following options, in your opinion what is the likely impact of artificial intelligence/machine learning on the work of GPs.**

No influence (GPs’ jobs will remain unchanged)
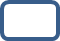
 _1_ Go to **Section C**

Minimal influence (GPs’ jobs will change slightly)
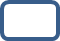
 _2_

Moderate influence (GPs’ jobs will change substantially)
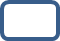
 _3_ First go to **B8**

Extreme influence (GPs’ jobs become obsolete)
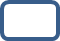
 _4_

**B8. Please briefly describe the way(s) you believe artificial intelligence/machine learning will change GPs’ jobs in the next 25 years.**

**
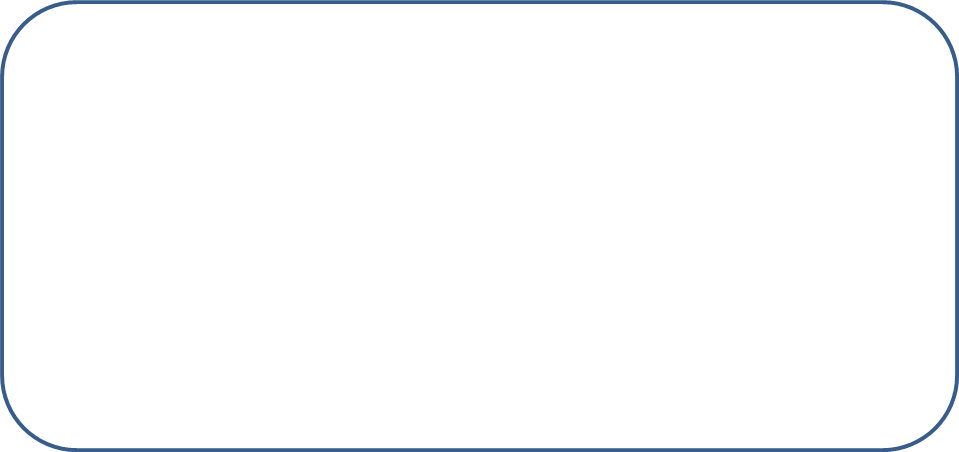
**

**Thank you for completing Section B.**

**Please turn over for Section C.**

**SECTION C**

**In this section we ask you to provide your level of agreement/disagreement with different statements.**

**C1. 25 years from now, technology (e.g., smartphone apps) will be used to decide when patients need to see a GP.**

Strongly disagree
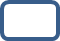
 _1_

Moderately disagree
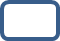
 _2_

Somewhat disagree
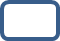
 _3_

Somewhat agree
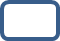
 _4_

Moderately agree
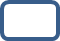
 _5_

Strongly agree
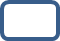
 _6_

**C2. 25 years from now, GPs will routinely work in partnership with artificial intelligence/machine learning to diagnose patients.**

Strongly disagree
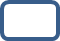
 _1_

Moderately disagree
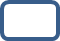
 _2_

Somewhat disagree
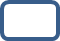
 _3_

Somewhat agree
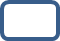
 _4_

Moderately agree
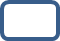
 _5_

Strongly agree
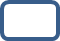
 _6_

**C3. 25 years from now, GPs will routinely work in partnership with artificial intelligence/machine learning to determine the likely course of a patient’s illness.**

Strongly disagree
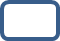
 _1_

Moderately disagree
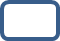
 _2_

Somewhat disagree _3_

Somewhat agree _4_

Moderately agree _5_

Strongly agree _6_

**C4. 25 years from now, GPs will routinely work in partnership with artificial intelligence/machine learning to devise patient treatment plans.**

Strongly disagree _1_

Moderately disagree _2_

Somewhat disagree _3_

Somewhat agree _4_

Moderately agree _5_

Strongly agree _6_

**C5. 25 years from now, remote monitoring of patients’ vital signs will be more common than in-person check-ups of vital signs with GPs.**

Strongly disagree _1_

Moderately disagree _2_

Somewhat disagree _3_

Somewhat agree _4_

Moderately agree _5_

Strongly agree _6_

**C6. 25 years from now, patients’ will have greater access to their own medical records than they do today.**

Strongly disagree _1_

Moderately disagree _2_

Somewhat disagree _3_

Somewhat agree _4_

Moderately agree _5_

Strongly agree _6_

**Thank you for completing Section C.**

**Please turn over for Section D.**

**SECTION D**

**This section asks you questions about the potential benefits and harms of artificial**

**intelligence/machine learning in medical care. Again, we ask you to provide your level of**

**agreement/disagreement with different statements.**

**D1. 25 years from now, the implementation of innovations in artificial intelligence/machine learning will reduce rates of diagnostic errors in medicine.**

Strongly disagree _1_

Moderately disagree _2_

Somewhat disagree _3_

Somewhat agree _4_

Moderately agree _5_

Strongly agree _6_

**D2. 25 years from now, the implementation of innovations in artificial intelligence/machine learning will increase patient access to medical care.**

Strongly disagree _1_

Moderately disagree _2_

Somewhat disagree _3_

Somewhat agree _4_

Moderately agree _5_

Strongly agree _6_

**D3. 25 years from now, the implementation of innovations in artificial intelligence/machine learning will reduce healthcare costs.**

Strongly disagree _1_

Moderately disagree _2_

Somewhat disagree _3_

Somewhat agree _4_

Moderately agree _5_

Strongly agree _6_

**D4. If offered the choice patients would prefer home monitoring of vital signs compared to visiting their GP.**

Strongly disagree _1_

Moderately disagree _2_

Somewhat disagree _3_

Somewhat agree _4_

Moderately agree _5_

Strongly agree _6_

**D5. Patients will always prefer receiving important medical information via face-to-face consultations with GPs rather than via their devices.**

Strongly disagree _1_

Moderately disagree _2_

Somewhat disagree _3_

Somewhat agree _4_

Moderately agree _5_

Strongly agree _6_

**D6. Please provide any brief comments you may have about the potential benefits of artificial intelligence/machine learning in medicine.**

**D7. Please provide any brief comments you may have about the potential harms of artificial intelligence/machine learning in medicine.**

**SECTION E**

**The questions in this section ask about your familiarity with artificial intelligence/machine learning.**

**E1. Have you heard of machine learning?**

No _1_

Yes _2_

**E2. Are you familiar with big data analytics?**

No _1_

Yes _2_

**E3. Have you read any academic journal articles about artificial intelligence/machine learning in medicine?**

No _1_

Yes _2_

**E4. Please estimate how many hours your instructors/lecturers *have spent* discussing artificial intelligence/machine learning during your medical degree so far.**

**E5. Please estimate how many hours your instructors/lecturers *will spend* discussing artificial intelligence/machine learning during the course of obtaining your medical degree.**

**E6. Do you plan to learn about artificial intelligence/machine learning as they pertain to medicine?**

No _1_

Yes _2_

Maybe _3_

**E7. Discussion about artificial intelligence/machine learning should be part of medical training.**

Strongly disagree _1_

Moderately disagree _2_

Somewhat disagree _3_

Somewhat agree _4_

Moderately agree _5_

Strongly agree _6_

**Thank you for completing Section E.**

**Please turn to the final question in Section F.**

**SECTION F**

**The question in this section is about your perceptions of the GP workforce.**

**F1. The number of primary care physicians worldwide is…**

Much less than the demand _1_

Somewhat less than the demand _2_

About equal to the demand _3_

Somewhat greater than the demand _4_

Much greater than the demand _5_

**THANK YOU FOR TAKING THE TIME TO COMPLETE THIS SURVEY!**

**PLEASE RETURN YOUR COMPLETED SURVEY**
